# CELEBRATING 30 YEARS OF ENHANCE FITNESS: REACHING NEW POPULATIONS OF OLDER ADULTS WITH EVIDENCE-BASED EXERCISE

**DOI:** 10.1093/geroni/igae098.0995

**Published:** 2024-12-31

**Authors:** Kushang Patel, Paige Denison, Steven Albert

**Affiliations:** Chair; Co-Chair; Discussant

## Abstract

EnhanceFitness® (EF) is an evidence-based, group exercise program for older adults involving strength, aerobic, and balance training that is recommended for arthritis management and falls prevention. Classes are held for 1 hour, 3 days a week and are led by exercise instructors trained in the EF protocol, which accommodates people with different levels of physical capacity. In the past 30 years, more than 150,000 older adults have participated in EF at >800 community sites in the United States. Over 75 peer-reviewed studies have evaluated EF’s effectiveness, implementation, and dissemination. In the proposed symposium, we will report new findings on efforts to expand EF’s reach. First, Paige Denison (who directs EF nationally) will present a brief overview of the program. Second, Kushang Patel will present the adaptation of EF for remote delivery (Tele-EF) and report the comparative effectiveness of Tele-EF versus in-person EF on several functional outcomes. Third, Nancy Gell will present a qualitative study on the experience of older rural cancer survivors with participation in Tele-EF. Fourth, Elise Hoffman will report the feasibility of Tele-EF in rural older adults with chronic pain and the results of co-designing a referral pathway to reach this population. Fifth, Basia Belza will present a pilot study on the feasibility of EF for people living with cognitive impairment and their caregivers. Lastly, Steven Albert (Editor of GSA’s Innovation in Aging) will deliver a critical discussion of the research findings and provide recommendations on expanding access of exercise programs to underserved, hard-to-reach populations of older adults. Fitness, Exercise and Wellness Interest Group Sponsored Symposium

